# Supplementary material for: Testing a first online intervention to reduce conformity to cyber aggression in messaging apps
Source: PLoS One. 2022 Aug 9;17(8):e0272615. doi: 10.1371/journal.pone.0272615 (PMC9362912; doi:10.1371/journal.pone.0272615)
Supplement: Attachment — Submitted filename: Response to Reviewers.docx [file pone.0272615.s001.docx]

March 24, 2022

Muhammad Shahzad Aslam, Ph.D., M.Phil., Pharm-D

Academic Editor

*PLOS ONE*

Subject: Revision of manuscript PONE-D-21-17000

Dear dr. Muhammad Shahzad Aslam and reviewers,

Please find enclosed our revised manuscript PONE-D-21-17000R1 entitled “Developing and testing an online behavior change intervention to reduce conformity to cyber aggression in messaging apps”. We greatly appreciate the time and effort that was dedicated to reviewing our manuscript. We thank dr. Muhammad Shahzad Aslam and the reviewers for their thorough review and comments, which were very helpful in improving our manuscript.

On the next pages, we specify how we have addressed the reviewers’ comments and we describe the changes we have made. In our response, the reviewers’ comments are numbered, and our responses follow below in blue. The revised manuscript text is displayed in italics, with page numbers for reference. Changes to the references list are indicated at the end of this response letter.

Thank you again for your consideration of our revised manuscript. We look forward to your response.

Sincerely, on behalf of all authors

Daniëlle N.M. Bleize

**Response to Reviewers’ Comments**

We were pleased to read that the reviewers valued our study and found the manuscript well-written. The reviewers suggest several revisions to improve our manuscript, which we address on the next pages.

**Reviewer #1**

*The manuscript is well written, contains detailed description of designed experiments, collected data, statistical evaluation and final explanations. I appreciate that the authors critically reviewed the results, including the fact that the initial hypotheses were not confirmed in the study. Such research is also necessary because it can raise new questions and lead to successive probably more detailed study.*

1. *The authors should add discussion about cultural influence. Would the results in another country be similar or different depending on cultural and social context?*

**Author response:** We thank the reviewer for this suggestion. Based on recent literature on the role of cultural influence in adolescents’ cyber aggression, we believe that our intervention might be more effective for adolescents from cultures with higher independent self-construal compared to adolescents from cultures with higher interdependent self-construal.

We have included two paragraphs in our discussion section that address the potential role of cultural influence and independent versus interdependent self-construals specifically. These paragraphs read as follows:

*“Finally, a fourth limitation is that our findings do not account for cultural influence. Recent research shows that cyber aggression is a worldwide phenomenon [57–59] and that the mechanisms involved in cyber aggression (such as social identification and reduced accountability) are mostly universal [59,60]. Yet, the prevalence of cyber aggression likely differs depending on specific learned cultural self-construals [57,58,60,61]. In European cultures for example, people typically have higher independent self-construal and view themselves as more separate from their social context. Contrarily, in many Asian cultures for example, people typically have higher interdependent self-construal and view themselves as more within a broader social context. Generally, higher independent self-construal is associated with higher involvement in cyber aggression perpetration [57,58,60,61]. Moreover, higher interdependent self-construal is associated with higher perception of social responsibility and better inhibitory control [62].*

*Because adolescents with higher independent self-construal are expected to be involved in cyber aggression more often and adolescents with higher interdependent self-construal are expected to be already better at inhibiting their behavioral responses, we would expect that the current intervention is more effective for adolescents with a higher independent self-construal. These adolescents may simply benefit most from an intervention helping them to better regulate their behaviors. Nevertheless, given that the intervention was not effective, we would not currently expect the results to be any different for adolescents with higher interdependent self-construal. However, our sample only included participants from the same (European) country. Therefore, we cannot actually examine the role of cultural influence with our own data. Our explanations of the potential effects of cultural influence are, thus, only speculative and remain of interest for future research.”* (p. 31-32, discussion)

**Reviewer #2**

*The papers presents a very current topic, regarding users online behaviour in messaging apps, where authors proposed an intervention using the Information-Motivation-Behavioral Skills model to counteract cyber aggression.*

1. *It is unclear how was applied the Information-Motivation-Behavioral Skills model and how it looks? Which is the mathematical model behind? Which are the variables of the model? The papers presents only the results of the IMB model, but the model description and implementation is lacking.*

**Author response:** We thank the reviewer for this remark. We used the Information-Motivation-Behavioral Skills model (IMB model) as a theoretical framework guiding the development of our intervention but did not explicitly test possible causal relationships between the variables of the IMB model. Therefore, we do not specify its mathematical model in our manuscript. However, we do agree with the reviewer that the description of the IMB model and how we implemented this in our intervention can be improved. To address the reviewer’s questions, we therefore made several changes or additions to the manuscript text.

First, we explicated that the IMB model principally offers a theoretical approach to develop behavioral interventions and that we used this theoretical approach to guide the development of our own intervention (p. 6-7). Second, we improved the general description of the IMB model by explaining the three key components of the model (information, motivation, and behavioral skills) and how these are interrelated (p. 6-7). Third, we added a figure (Fig 1) of the IMB model as applied to the concepts in our study, with the hope that this better explains how the key components of the IMB model translate to the specific topic of conformity to cyber aggression in our intervention. This figure is included with our resubmission, is referenced in the manuscript text (p. 7), and is displayed below. Finally, we more explicitly described how each component of the IMB model was implemented in our intervention by rephrasing sections throughout the manuscript (p. 7, 11-16, 20).

Revisions were made throughout the sections ‘Systematically translating theoretical insights to an applied intervention’, and ‘Testing a behavior change intervention to reduce conformity in messaging apps’ in the introduction section, and throughout the method section (visible in the revised manuscript with changes and indicated with the references to page numbers above).

**Fig 1. Information-Motivation-Behavioral Skills Model Applied to Conformity to Cyber Aggression.**

2. *The Conclusion and implications section can be integrated together with study limitations and future research directions. The whole effort seems useless if in Conclusions the authors stated that "the intervention was not successful in reducing conformity to cyber aggression, our study provides important insights and recommendations for the development of future intervention efforts."*

**Author response:** As per suggestion of the reviewer, we have integrated the two sections ‘Limitations and future research directions’ and ‘Conclusion and implications’ into one section named ‘Limitations, future research directions, and implications’. We rephrased this section of the discussion, in particular the final paragraph stating the implications of our study. We feel that this paragraph now better reflects how our study contributes to research and practice considering its limitations and suggested directions for future research. The final paragraph now reads as follows:

*“In conclusion, the findings of this study point to the complex relation between theory and practice: Although the theoretical underpinnings of desired behavior may be evident, manipulating these in an applied intervention can be challenging. Our study was a first effort towards doing so and its limitations and suggested research directions are valuable in informing future intervention efforts to reduce conformity to cyber aggressive behaviors in adolescents’ messaging apps.”* (p. 33, discussion)

**Changes to the Reference List**

The following references were added to the reference list (no other changes were made):

57.	Barlett CP, Gentile DA, Anderson CA, Suzuki K, Sakamoto A, Yamaoka A, et al. Cross-cultural differences in cyberbullying behavior: A short-term longitudinal study. J Cross Cult Psychol. 2014;45(2):300–13.

58.	Leung ANM, Wong N, Farver JM. Testing the effectiveness of an e-course to combat cyberbullying. Cyberpsychology, Behav Soc Netw. 2019;22(9):569–77.

59.	Kowalski R, Limber SP, McCord A. A developmental approach to cyberbullying: Prevalence and protective factors. Aggress Violent Behav. 2019;45(March–April):20–32.

60.	Barlett CP, Seyfert LW, Simmers MM, Hsueh Hua Chen V, Cavalcanti JG, Krahé B, et al. Cross-cultural similarities and differences in the theoretical predictors of cyberbullying perpetration: Results from a seven-country study. Aggress Behav. 2021;47(1):111–9.

61.	Wright MF, Kamble S V., Soudi SP. Indian adolescents’ cyber aggression involvement and cultural values: The moderation of peer attachment. Sch Psychol Int. 2015;36(4):410–27.

62.	Shapka JD, Law DM. Does one size fit all? Ethnic differences in parenting behaviors and motivations for adolescent engagement in cyberbullying. J Youth Adolesc. 2013;42(5):723–38.
