# Supplementary material for: Testing a first online intervention to reduce conformity to cyber aggression in messaging apps
Source: PLoS One. 2022 Aug 9;17(8):e0272615. doi: 10.1371/journal.pone.0272615 (PMC9362912; doi:10.1371/journal.pone.0272615)
Supplement: Attachment — Submitted filename: Response to Editor Comments.docx [file pone.0272615.s002.docx]

June 10, 2022

Muhammad Shahzad Aslam, Ph.D., M.Phil., Pharm-D

Academic Editor

*PLOS ONE*

Subject: Revision of manuscript PONE-D-21-17000R1

Dear dr. Muhammad Shahzad,

Please find enclosed our revised manuscript PONE-D-21-17000R1 entitled “Developing and testing an online behavior change intervention to reduce conformity to cyber aggression in messaging apps”.

We appreciate the time and effort that you invested as academic editor to review our manuscript. We thank you for your additional comments to our manuscript. We believe these have helped us greatly in improving our manuscript. Also, we would like to express our gratitude for granting us sufficient time to work on this revision.

On the next pages, we outline how we have addressed the comments and we describe the changes we have made. In our response, the comments are numbered and our responses follow below (in blue). The revised manuscript text is in italics, with page numbers for reference. Changes to the reference list are indicated at the end of this response letter.

Thank you again for your consideration of our revised manuscript. We look forward to your response.

Sincerely, also on behalf of my co-authors,

Daniëlle N.M. Bleize

**Response to Editor Comments**

1. Kindly write the abstract in a structured manner such as background, objective, methodology, results and conclusion. No need to give these headings but the abstract must be comprehensive.

**Author response:** We thank the editor for this comment. We agree that the abstract was not fully comprehensive, particularly with regards to the methodology of our study. We have critically revised the abstract and believe that it now provides a better structured overview of our study. Following the editor's comment, we did not include headings in the abstract. However, we rephrased sections of the abstract to more explicitly indicate its structure. For example, we now explicitly state the objective of our study ("…*the objective of the current preregistered study was to*…", p.2), outline the study procedure ("…*participants were first informed about*…", p. 2), and include the analysis we used to examine our main hypotheses ("*Factorial ANCOVAs revealed that*…", p. 2). Moreover, we extended the methodology section of the abstract. We believe that the abstract (p. 2 of the manuscript) now more clearly explains how the intervention was developed and how the IMB model was implemented.

2. Please provide the heading of the theoretical background and give complete literature in context to the theoretical background. Please provide all hypothesis related to Information-Motivation-Behavioral Skills Model Applied to Conformity to Cyber Aggression. (Fig 1)

**Author response:** Following the editor's suggestion, we added a heading for the theoretical background. We revised the introduction and theoretical background and revisited the literature. We conducted a literature search to search for (recent) relevant articles that we might have missed in the previous version of our manuscript. Based on this, we added recent articles as well as some key literature to the theoretical background. Moreover, we extended the explanation of recent empirical findings in the theoretical background (for example regarding our previous experiments, on p. 7 of the manuscript).

After careful consideration, we have decided to omit the figure of the IMB model (previously Fig 1) from our manuscript. Fig 1 displayed the IMB-model applied to conformity to cyber aggression. However, we believe that this figure may cause confusion among the reader regarding the objective and design of our study, which we would like to prevent. We designed this intervention based on the IMB principles (that adolescents should be informed, motivated and able to perform behavior). We compared different versions of the intervention and all versions targeted all IMB elements. It was not our goal to measure or actually test the paths of the IMB framework itself. That is, we did not aim to examine the (inter)relations between the IMB elements. Rather, we aimed to compare the presence of such elements in an intervention with the absence of them (control condition). Therefore, we did not have any hypotheses about these relations. Moreover, we also lack the necessary measures to actually test these (inter)relations. We only measured perceived importance and ease to conform to be able to explore how the intervention was evaluated. These served more or less as proxy's for motivation and behavioral skills, but we did not include proper measures for these variables and included no measure for the information element. To prevent confusion regarding our study goals, we therefore consider it best to omit Fig 1 from the manuscript. We revised the description of the IMB framework and its implementation (p. 8-10, 15-20, and 25) to better clarify that we used the IMB framework to guide the development of the intervention conditions, but not to test the paths in the IMB framework itself.

3. The abbreviation such as T1 and T2 is meaningless. Please revisit the abbreviations. If you want to use these then please illustrate them in a picture.

**Author response:** We acknowledge that the abbreviations were not meaningful in the previous version of our manuscript, because we did not specify their meaning before using them. Following the editors' comment, we decided to remove these abbreviations and instead use the terms 'Time 1' and 'Time 2'. In our manuscript we specify that 'Time 1' and 'Time 2' refer to the measurement times directly after the intervention and four weeks post-intervention respectively. We have also adapted these abbreviations in Fig 1 and Fig 2.

4. Please provide an illustration of the study design incomplete manner.

**Author response:** We have revisited all figures submitted with our manuscript, including the figure that displays our study design. We have created a new illustration that displays the study design as well as the study procedure that participants followed. The illustration displays the steps we took in preparing the study, and the two measurement sessions (Time 1 and Time 2) with their respective steps (e.g., questionnaires, intervention, messaging app paradigm). This illustration is displayed in Fig 1 (referred to on p. 15 of the manuscript and enclosed separately with our submission).

5. Please write the analysis name instead to use the term additional analysis OR Main analyses.

**Author response:** Following the editor's suggestion, we changed the headings of the analyses paragraphs in the results section of the manuscript (p. 30 and p. 33). The headings now include the name of the analysis that we conducted. We also identified any mention of the terms 'additional analysis' and 'main analysis' and replaced these with the name of the actual analysis that was used.

6. Please write the manuscript in a simple way. The manuscript was prepared in a complicated manner and was difficult to read and understand the analysis. Please revisit your figures and provide clear information.

**Author response:** We thank the editor for this comment. We have thoroughly rewritten the entire manuscript (see document Revised Manuscript with Track Changes). Some important changes we made include: (1) rewriting sentences from passive voice to active voice, (2) simplifying sentences by converting overly long sentences to shorter sentences, and (3) correcting for grammar and punctuation. In addition, we made many other changes throughout the paper to help the reader's understanding of our manuscript. For example, we revised the results section of the manuscript to clarify our findings. We hope that the manuscript now reads more easily and that the analyses are more clear. Finally, we have revisited our figures. This led to a new illustration for the study design and study procedure (Fig 1) and an updated illustration for the charts that display the means for the outcome variables per condition (Fig 2).

**Changes to the Reference List**

The following references were added to the reference list:

Cialdini RB, Goldstein NJ. Social influence: Compliance and conformity. Annu Rev Psychol.	2004;55:591–621.

Gardella JH, Fisher BW, Teurbe-Tolon AR. A systematic review and meta-analysis of cyber-	victimization and educational outcomes for adolescents. Rev Educ Res. 2017;87(2):283–308.

Giletta M, Choukas-Bradley S, Maes M, Linthicum KP, Card NA, Prinstein MJ. A meta-analysis of	longitudinal peer influence effects in childhood and adolescence. Psychol Bull.	2021;147(7):719–47.

Giumetti GW, Kowalski RM. Cyberbullying via social media and well-being. Curr Opin Psychol	[Internet]. 2022;45:101314.

Hinduja S, Patchin JW. 2019 Cyberbullying data [Internet]. 2019. Available from:	https://cyberbullying.org/2019-cyberbullying-data

Laursen B, Veenstra R. Toward understanding the functions of peer influence: A summary and	synthesis of recent empirical research. J Res Adolesc. 2021;31(4):889–907.

Lozano-Blasco R, Cortés-Pascual A, Latorre-Martínez MP. Being a cybervictim and a cyberbully – The	duality of cyberbullying: A meta-analysis. Comput Human Behav. 2020;111:106444.

Nguyen HTL, Nakamura K, Seino K, Vo VT. Relationships among cyberbullying, parental attitudes, self-	harm and suicidal behavior among adolescents: Results from a school-based survey in	Vietnam. BMC Public Health. 2020;20(1):1–10.

Olthof T, Goossens FA. Bullying and the need to belong: Early adolescents’ bullying-related behavior	and the acceptance they desire and receive from particular classmates. Soc Dev.	2008;17(1):24–46.

Rudnicki K, Vandebosch H, Voué P, Poels K. Systematic review of determinants and consequences of	bystander interventions in online hate and cyberbullying among adults. Behav Inf Technol	[Internet]. 2022;1–18.

Skilbred-Fjeld S, Reme SE, Mossige S. Cyberbullying involvement and mental health problems among	late adolescents. Cyberpsychology. 2020;14(1).

Steer OL, Betts LR, Baguley T, Binder JF. “I feel like everyone does it”- adolescents’ perceptions and	awareness of the association between humour, banter, and cyberbullying. Comput Human	Behav [Internet]. 2020;108:106297.

Wegge D, Vandebosch H, Eggermont S, Pabian S. Popularity through online harm: The longitudinal	associations between cyberbullying and sociometric status in early adolescence. J Early	Adolesc. 2016;36(1):86–107.
