# Supplementary material for: Testing a first online intervention to reduce conformity to cyber aggression in messaging apps
Source: PLoS One. 2022 Aug 9;17(8):e0272615. doi: 10.1371/journal.pone.0272615 (PMC9362912; doi:10.1371/journal.pone.0272615)
Supplement: Attachment — Submitted filename: Response to Reviewer Comments.docx [file pone.0272615.s003.docx]

July 13, 2022

Muhammad Shahzad Aslam, Ph.D., M.Phil., Pharm-D

Academic Editor

*PLOS ONE*

Subject: Revision of manuscript PONE-D-21-17000R2

Dear dr. Muhammad Shahzad Aslam,

Thank you for giving us the opportunity to submit a revised draft of our manuscript PONE-D-21-17000R3 entitled “Testing a first online intervention to reduce conformity to cyber aggression in messaging apps” for publication in *PLOS ONE*.

We want to express our gratitude for the time and effort that you and the reviewers dedicated to providing feedback on our manuscript. We thank you for the insightful comments and suggestions for improvement to our manuscript. We have incorporated all of the suggestions made by the reviewers.

On the next pages, we outline how we have addressed the suggestions and describe the changes that we made. In our response, the comments are numbered and our responses follow below (in blue). The revised manuscript text is in italics, with page numbers for reference (referring to the submitted document 'Manuscript'). Changes to the reference list are indicated on the final page of this response letter.

Thank you again for your consideration of our revised manuscript. We look forward to your response.

Sincerely, also on behalf of my co-authors,

Daniëlle N.M. Bleize

**Reviewers' Comments to the Authors:**

**Reviewer 3**

1. In the abstract, this phrase “Our findings point to the complex relation between theory and practice and warrant future research to further examine the effectiveness of intervention tools to reduce conformity to cyber aggression” will make me confused about the meaning. Or it can be revised as “. Our findings point to the complex relation between theory and practice, and these can warrant future research to further examine the effectiveness of intervention tools which could be used to reduce the conformity of cyber aggression and behavioral intervention tools”. Please revised this article again and make it more readable.

**Author response:** As suggested by the reviewer, we have revised this phrase in the abstract. The phrase now reads:

"*The findings from this first intervention effort point to the complex relationship between theory and practice. Our findings warrant future research to develop potential intervention tools that could effectively reduce conformity to cyber aggression*." (p. 2)

Moreover, we agree with the reviewer's assessment that we can further improve the readability of our manuscript. Accordingly, throughout the manuscript, we made revisions to the text to make it more readable. The revisions include shortening and improving brevity of the manuscript text, eliminating passive voice, clarifications and many other things. We do not include all revisions in this response letter (because they are too many), but the revisions are visible in the submitted document titled 'Revised Manuscript with Track Changes'.

2. Please draw these two pictures again, because these two are so blurred. The font should be revised to bigger one.

**Author response:** Thank you for pointing this out. As suggested by the reviewer, we have redrawn both figures with a bigger font and improved image quality.

3. For the Limitation and future research direction part, the limitations are described so readable and obvious, but the future work and feasibility need more details. Especially, for the third limitation, if you could not find the proper participants, is there any alternative plan, for example, collecting data from the third-world countries, or using some mental health scales to avoid aggression trend?

**Author response:** Thank you for pointing this out. The reviewer is correct that we did not adequately describe the future research directions for the third and fourth limitation. As suggested by the reviewer, we have added the following to the manuscript text to describe the future research directions related to the third limitation (that concerns the possibility of sampling bias):

"*Future research should aim to draw representative samples from the population that include both adolescents with little or no involvement in cyber aggression and those who were previously a perpetrator or a victim. Research with such representative samples is important, because this might shed light on the effectiveness of interventions for different target populations. Moreover, it would allow for the development of more tailored interventions, where interventions fit participants' personal characteristics or previous experiences such as cyber aggression involvement [71]. Some research suggests that tailored interventions can be more successful in changing behavior compared to generic interventions [72]. Obtaining a more representative sample could be achieved by informing parents and participants more explicitly that they do not have to share any personal experiences and that their anonymity is guaranteed*." (p. 31)

Furthermore, we have added the following to the manuscript text to describe the future research directions related to the fourth limitation (that concerns the lack of cross-cultural comparisons):

"*We recommend future research on conformity to cyber aggression in messaging apps to broaden the scope and compare the effectiveness of interventions cross-culturally. For example, data could be collected and compared in countries with interdependent self-construal cultures (e.g., Egypt, Pakistan, Japan) and independent self-construal cultures (e.g., the United States, Australia, Germany). Comparing these countries could shed light on the most effective intervention design for adolescents with different cultural backgrounds*." (p. 32)

4. In the Conclusion, please attach the p level after the phrase, when you are summarizing the findings. This would become more readable.

**Author response:** As suggested by the reviewer, we have added the p-levels after the relevant phrases in the conclusion (p. 28).

5. Considering to change the title. “Developing and testing an online behavior change intervention to reduce conformity to cyber aggression in messaging apps” would make readers think that this article can give them a practical and useful way to address cyber aggression.

**Author response:** We think this is an excellent suggestion. We have changed the manuscript title to: "Testing a first online intervention to reduce conformity to cyber aggression in messaging apps". We believe that this title better reflects that we test the effectiveness of a first intervention effort, without implying that this effort was effective and immediately implementable in practice.

**Changes to the Reference List**

The following references were added to the reference list:

Jacobs NC, Völlink T, Dehue F, Lechner L. Online Pestkoppenstoppen: Systematic and theory-based	development of a web-based tailored intervention for adolescent cyberbully victims to	combat and prevent cyberbullying. BMC Public Health. 2014;14(396):1–16.

Krebs P, Prochaska JO, Rossi JS. A meta-analysis of computer-tailored interventions for health	behavior change. Prev Med (Baltim). 2010;51(3–4):214–21.
